# Bibliometric Analysis of Research on the Main Genes Involved in Meat Tenderness

**DOI:** 10.3390/ani12212976

**Published:** 2022-10-29

**Authors:** Jhony Alberto Gonzales-Malca, Vicente Amirpasha Tirado-Kulieva, María Santos Abanto-López, William Lorenzo Aldana-Juárez, Claudia Mabel Palacios-Zapata

**Affiliations:** 1 Laboratorio de Tecnología de Alimentos y Procesos, Universidad Nacional de Frontera, Sullana 20100, Peru; 2 Laboratorio de Biología Molecular, Universidad Nacional de Frontera, Sullana 20100, Peru; 3 Facultad de Ingeniería de Industrias Alimentarias y Biotecnología, Universidad Nacional de Frontera, Sullana 20100, Peru

**Keywords:** meat tenderness, genes, calpain system, CAPN, CAST, SNP, marker-assisted selection, genomic selection, bibliometric study

## Abstract

**Simple Summary:**

A bibliometric analysis was carried out to know the evolution of research on genes associated with meat tenderness, of interest for the development of selection programs. Since 1993, studies have been limited to a few researchers in high-income countries due to the costs associated with the techniques. The main findings showed that the scientific production had a discontinuous growth because science experienced a significant change since approximately 2010. Marker-assisted selection was widely used, evaluating mainly CAPN (calpain) and CAST (calpastatin) genes for their contribution to meat tenderness, especially in cattle. However, the effects are small; therefore, genomic selection was implemented by genotyping thousands of single nucleotide polymorphisms (SNPs) for further explanation of genetic variation. The results shown are important for scholars to identify emerging methodologies and gaps in the literature and to know who the prolific authors and institutions in the field for possible collaborations, etc., are.

**Abstract:**

Tenderness is one of the main characteristics of meat because it determines its price and acceptability. This is the first bibliometric study on the trend of research on the role of genes in meat tenderness. A total of 175 original and English-language articles published up to 2021 were retrieved from Scopus. The bibliometric analysis was carried out with VOSviewer (version 1.6.18, Eck and Waltman, Leiden, Netherlands) and complemented with the Analyze search results service from Scopus. Erroneous and duplicate data were eliminated, and incomplete information was added to standardize the results. Scientific production was evaluated by means of quantity, quality and structure indicators. As a first glance, 8.816% of authors have published more than 50% of papers mainly related to genes encoding the calpain (CAPN)-calpastatin (CAST) system and single nucleotide polymorphisms (SNPs). Among other findings, a strong link was found between the contribution of the main countries (led by the United States with) and their institutions (led by the USDA Agricultural Research Service with) to their gross domestic product. Most studies on the topic are published in the *Journal of Animal Science*, and other journals with high impact according to the number of citations and different metrics. Finally, when evaluating the most cited articles, the occurrence and association of the main keywords, it was confirmed that research is focused on the role of CAPN and CAST genes and of SNPs in beef tenderness. The change in science was emphasized; although marker-assisted selection is still used, genes have an infinitesimal effect on complex traits. Therefore, since about 2010, new research groups adopted genomic selection to evaluate dense panels of SNPs and better explain genetic variation in meat tenderness.

## 1. Introduction

Meat is an important food for human health due to its nutritional characteristics; its world production in 2020 exceeded 413 million tons [1]. Consumers today demand that foods meet the highest quality standards. This is a challenge for meat producers and processors [2]. Among the meat quality attributes, scientists have focused on the study of color, tenderness, juiciness and water-holding capacity because of their influence on consumer acceptability [3]. Tenderness is considered one of the main palatability factors, especially for red meat [4]. Tender meat satisfies consumer demand and increases purchase frequency and willingness to pay higher prices [2,5]. Ellies-Oury et al. [6] evaluated the opinion of French people on beef quality. They found that 88% of the respondents stated that the level of tenderness and flavor of the meat should be guaranteed at the time of purchase. In China, Kantono et al. [7] found that 40.72% of respondents prioritized tenderness when purchasing lamb meat. In a study conducted in the United States, 78% of consumers evaluated were willing to buy and pay more for tender steaks [8]. In the same country, it was determined that the variability in beef tenderness decreases its value by more than USD 7 per animal and more than USD 200 million of unattained revenue for the industry [9].

The underlying mechanisms of meat tenderization have been the subject of research for several decades [10]. During aging, variation in meat tenderness was shown to depend on muscle myofibril proteolysis, intramuscular fat, muscle composition, sarcomere length and connective tissue content [11,12,13,14,15].

The evaluation of meat tenderness by expert panelists is difficult, costly and subjective and cannot be measured until after the animals are harvested. However, the high potential of gene identification (markers) to predict meat quality has been reported as suitable for management and selection programs [16]. There is evidence that multiple genes control meat quality characteristics [5]. Smith et al. [17] determined that meat tenderness is defined by genetic and environmental factors in proportions of 46 and 54%, respectively. In general, genes have an infinitesimal effect on complex traits; consequently, selection can be based on the total expected effect of the genes of interest [18]. Therefore, it is necessary to identify the evolution and/or changes in the methodologies for the selection and crossing of animals with better tenderness traits.

Currently, the presence of millions of research articles available online makes it difficult to retrieve and analyze relevant papers [19]. Comprehensive reviews [2,5,12,15,20,21,22,23,24,25,26] and systematic reviews [20,27] were conducted on the study of genes involved in meat quality (tenderness in particular). These reviews provided valuable information, but additional insight is needed to inform progress and trends in the field. Huertas-Valdivia et al. [28] suggest scientific mapping using bibliometric methods that show the structure and dynamic aspects of cumulative knowledge. Bibliometrics is the evaluation of large volumes of scientific data [29] to answer questions such as: How many papers have been published annually? Which authors, countries and institutions contribute the most research in the field of study? Which are the main journals in which research is published? Which are the most cited articles? What is the trend in keyword usage? [30]. Scholars can use the results to evaluate the evolution of science, determine outdated and emerging methodologies, identify and explore a gap in the literature, solicit support from prolific authors in a field, conduct research in countries with little knowledge and production on the topic of study, select relevant papers (based on the number of citations) to start a new study, etc. Bibliometric analysis is quantitative, rigorous and objective, allowing for a systematic, transparent and reproducible study [30]; therefore, it is more efficient than a traditional literature review [31].

To our knowledge, this study will report the research trends and knowledge gaps on the role of genes in meat tenderness through a bibliometric analysis for the first time.

## 2. Materials and Methods

In September 2022, a document search was performed in the Scopus database (Elsevier B.V., Amsterdam, The Netherlands) because of its large number of abstracts and citations of peer-reviewed literature [32]. In addition, Scopus provides a data analysis tool to complement the bibliometric evaluation. To obtain more accurate and reliable results, the document search was performed by article title, abstract and keywords [31] with the following string: (“meat tenderness” AND gene). Only original articles in English were included to avoid duplicates, and only articles published up to 2021 were included to compare complete annual periods [32].

The information was exported in CSV format, and the bibliometric analysis was performed with VOSviewer (version 1.6.18, created by Eck and Waltman, Leiden, The Netherlands), a widely used free software [33]. To complement the analysis, the Analyze search results service from Scopus, which provides data of interest such as the number of papers per year, number of citations per paper, number of papers per author, per affiliation, per country, and per subject area, was used. However, databases such as Scopus are not designed for bibliometric analysis. For example, an author’s profile may show more than one affiliation (due to changes of institution over time), which is reflected in the data obtained; however, only the affiliation or affiliations corresponding to each paper published should be left [33]. Another error can be shown in the author’s name. Taking as an example an author of this study (Vicente Amirpasha Tirado-Kulieva), the information may reflect the papers published by Tirado-Kulieva, V. A. and those published by Kulieva, V. A. T. (if “-” is omitted between Tirado and Kulieva), although he is the same author. In this regard, to ensure a consistent and standardized data set, all authors reviewed the information (data were added/deleted as appropriate) prior to analysis.

To evaluate scientific production, the three bibliometric indicators proposed by Durieux and Gavenois [34] were considered:

(a) Quantity or productivity indicator: number of papers by main subject areas, authors, countries, institutions and journals.
(b) Quality or performance indicator: number of citations for the aforementioned elements, in addition to some metrics that measure the impact of the journals.
(c) Structural or connections indicator: collaboration maps between authors and between countries, grouping of journals and keywords.

## 3. Results and Discussion

### 3.1. Publications by Subject Area

**Figure 1:**
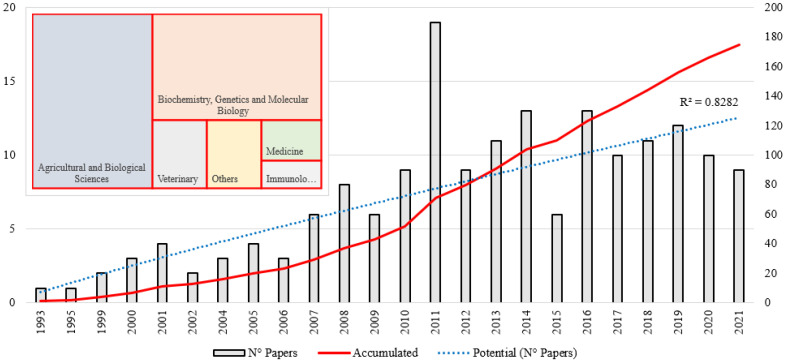
Papers published on the role of genetics in meat tenderness.

As shown in Figure 1, for the period 1993–2021 there is a cumulative 175 papers published. In 1993 (1 paper), research focused on identifying genes associated with meat tenderness began, but this boom only lasted about two decades with its peak in 2011 (19 papers, cumulative: 71 papers). This milestone is related to the implementation of genomic selection in the livestock industry. Previously, selection for economically important traits was performed by marker-assisted selection (MAS), which has contributed significantly in the field. However, MAS employs a small number of DNA markers to recognize a limited number of quantitative trait loci (QTL) with infinitesimal effects for complex traits whose genetic variation is explained by many QTL [35]. This prompted the development of a new methodology called genomic selection [36], which was shown to be a promising alternative to accelerate genetic improvement by evaluating dense panels of single nucleotide polymorphisms (SNPs) that explain most of the genetic variation in important traits [37]. Around 2010, the implementation of genomic selection considerably changed the cattle industry, improving meat production [38]. Progress occurred gradually thanks to the release of SNP dense chips (arrays) such as ParAllele 10K (Affymetrix Inc., Santa Clara, CA, USA) in 2005, followed by Bovine SNP50 BeadChip, in addition to other genotyping chips released in 2010 such as Bovine3K and BovineHD (all three from Illumina Inc., San Diego, CA, USA), all for cattle [39]. The number of papers published subsequently decreased and remained relatively constant until 2021 (9 papers). Even at the time of the search, there were 10 papers published in 2022. It appears that the new methodology in science has been established. To define the evolution of the field of genetics involved in meat tenderness, we consider the classification of Derek John de Solla Price, considered the father of scientometrics. Price [40] established that the development of a discipline in general begins with a base (precursor) stage; when there is a significant amount of information, exponential growth begins; finally, growth continues, but slowly (linear). The power trend line (R^2^: 0.8282) is the one that best fits the number of papers per year, i.e., publications are made at a specific rate. As mentioned, this discontinuous evolution in science since about the last decade is due to changes in studies on the genetics of meat tenderness.

Figure 1 also shows the five main subject areas of the studies. Since a paper may be in several categories, it is not appropriate to show the number of papers per area; therefore, a rectangular chart was created to hierarchize the data based on their proportion. Agricultural and biological sciences (40.909%) is the main category, followed by biochemistry, genetics and molecular biology (33.566%), veterinary (9.091%), medicine (3.846%), immunology and microbiology (3.147%). The other subject areas (9.441%) are chemical engineering, engineering, multidisciplinary, chemistry, computer science, nursing and social sciences.

### 3.2. Main Authors, Countries, Affiliations and Journals

The 10 authors, countries, affiliations and journals with the most contribution to the field of study were defined according to the number of papers. In cases of equivalence, the ranking was defined by the number of citations, which indicates the relative influence and importance [41]. It is emphasized that the number of papers and citations indicate the relevance of a topic, not the quality of the paper itself [42].

#### 3.2.1. Most Prolific Authors

All the authors shown in Table 1 have been published for about two decades and remain (relatively) current in the field, with the exception of Keele, J. W. (1999–2005). Smith, T. P. L. is the author with the most research (13 papers, 7.429% of total) on the influence of genes on meat tenderness. His most recent paper was published by Bennet et al. [43]. They determined that CAPN1 (encoding µ-calpain or calpain 1) and MSTN (encoding myostatin) gene polymorphisms play a role in beef tenderness. In this article, Smith, T.P.L. shares authorship with Casas, E.; Shackelford, S. D.; Wheeler, T. L.; and Bennet, G. L., who are also ranked with 12, 10, 8 and 7 papers published, respectively. Regarding the other prolific authors, the most recent paper by de Oliveira H. N. (11 papers) used a genomic selection (genotyping with panels containing 74,677 and 777,962 SNPs) and estimates of the heritability of different phenotypic traits in Nelore cattle. Tenderness in longissimus thoracis was one of the traits with the highest accuracy (up to 0.60) [44]. In the most recent article by Koohmarie, M. (11 papers), different genetic and biochemical parameters related to tenderness were evaluated in longissimus muscles of Bos indicus and B. taurus crosses, highlighting the role of CAST (encoding calpastatin) and CAPN genes [45]. In the latest paper by Keele, J. W. (11 papers), the CAPN1 gene and its various SNPs showed a significant and positive relationship with tenderness in B. taurus and B. indicus [46]. In the last study published by Chardulo, L. A. L. (9 papers), myosin (MyH) gene expression showed a relationship (positive or negative depending on the isoform) with tenderness in longissimus thoracis of Nelore cattle [47]. Finally, the most recent paper by Coutinho, L. L. (8 papers) identified quantitative trait loci of muscle allele-specific expression that affect traits such as tenderness in longissimus thoracis of B. indicus [48]. Up to this point, most studies related to meat tenderness focus on genes encoding the calpain–calpastatin system and SNPs.

The number of citations is also a key parameter that defines the relevance or influence of the paper in the field of study. Tahantam et al. [49] retrieved 198 relevant papers from Scopus, Web of Science, PubMed and Medline and determined that the number of citations depends on factors related to the paper, but also on factors related to the journal and the author. The citations (TC) of each author depend on the number of papers published (TP). For comparison purposes, the number of papers/(TC/TP) ratio of each author is as follows: Smith, T. P. L. 1:6; Casas; E. 2:6; de Oliveira, H. N. 3:10; Koohmaraie, M. 4:2; Keele, J. W. 5:5; Shackelford, S. D. 6:4; Chardulo, L. A. L. 7:9; Wheeler, T. L. 8:3; Coutinho, L. L. 9:8 and Bennett, G. L. 10:7. The change in the ranking was substantial; the TC/TP is a determining factor in the ranking.

Price [50] proposed a way to determine the number of main authors and papers published in a field according to Equation 1; m is the minimum number of papers to define the main authors, and n_max_ is the largest number of papers by an author (13 by Smith T. P. L.). The m value is 2.7 (rounded to 3); there are 73 main authors with at least 3 papers, giving a total of 102 different papers. Considering the 828 authors of the 175 papers, 8.816% of authors have contributed 58.256% of papers on research on genes associated with in meat tenderness.

(1) $$m\;=0.749\times \sqrt{n_{\max}}$$

The 175 papers involved 828 authors. More than 50% (52.571%) of the papers were published with more than five authors. There is 1 paper (0.571%) with only 1 author, 8 papers (4.571%) with 2 authors, 15 papers with 3 authors (8.571%), 16 papers (9.143%) with 4 authors, 22 papers (12.571%) with 5 authors, and 21 papers (12%) with more than 10 authors. The average number of authors per paper is 4.81, i.e., studies on the topic are usually conducted collaboratively [51].

**Figure 2:**
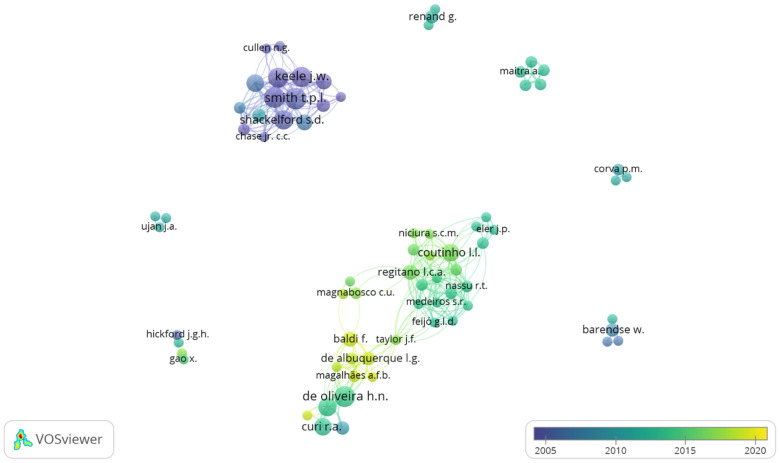
Overlay visualization network of the top 73 authors in the field.

Co-authorships are shown in Figure 2 by an overlay visualization network whose elements (authors, in this case) are labeled in colored circles and connected with lines. The higher the frequency (number of papers), the larger the circle and label; the colors of the circles distinguish the time at which the authors published their papers, and the distance between each element represents their correlation [31].

Of the 73 main authors defined above, more than five well-defined clusters can be seen. The most representative group is formed by Wheeler, T. L.; Casas, E.; Smith, T. P. L.; Shackelford, S. D.; Keele, J. W.; Koohmaraie, M.; etc., who are among the authors with the greatest contribution to the field (Table 1). However, according to the timeline, these authors have not contributed since approximately 2010. In the period 2012–2016 the group comprising de Coutinho, L. L. L.; Feijó, G. I. D.; Regitano, L. C. A.; Nassu, R. T.; Magnabosco, C. U.; Medeiros, S. R.; Niciura, S. C. M.; etc., published its papers. In addition six small groups represented by Renand, G. Maitra, A.; Corva, P. M.; Barendse, W.; Ujan, J. A.; Gao, X. Later, the group formed by Baldi, F.; de Albuquerque L. G.; Curi, R. A.; Magalhães, A. F. B.; Taylor, J. F.; de Oliveira, H. N.; etc., contributed publications from 2010 to 2019.

Most studies on the evaluation of different genes (LEP, HSP90AA1, FABP4, GH1, HSPB1, MYF5, MYF5, DNAJA1, DGAT1, HRSP12, ADAMTS4, etc., mainly CAPN, CAST and their isoforms) and their SNPs as markers for tenderness traits were performed until about 2010 (e.g., [45,46,52,53,54,55,56,57,58,59,60,61,62,63,64,65,66]), but later, related papers were also published on cattle of different breeds and in other geographic locations [67,68,69,70,71,72,73,74,75,76]. The new research groups are especially focused on the use and improvement of genomic selection [32,44,77,78,79,80,81] and also on the use of RNA sequencing technology to identify the mechanisms involved in gene expression for beef tenderness [82,83,84].

#### 3.2.2. Most Prolific Countries

The United States contributed more papers (41), but New Zealand has a higher TC/TP ratio (46.500). The number of papers/(TC/TP) ratio of the countries shown in Table 2 is as follows: United States 1:2, Brazil 2:5, China 3:10, Australia 4:4, Spain 5:9, France 6:3, South Korea 7:8, New Zealand 8:1, Canada 9:8 and Poland 10:6. The evaluation of the association between genes and meat tenderness is part of biotechnological studies, and its interest and depth depends on the gross domestic product (GDP) of the countries [85]. The relationship between the number of papers of the countries shown in Table 2 and their GDP is as follows: United States 1:2, Brazil 2:3, China 3:1, Australia 4:8, Spain 5:6, France 6:4, South Korea 7:5, New Zealand 8:10, Canada 9:7 and Poland 10:9 [86]. In this case, the variation between each classification is slight.

**Figure 3:**
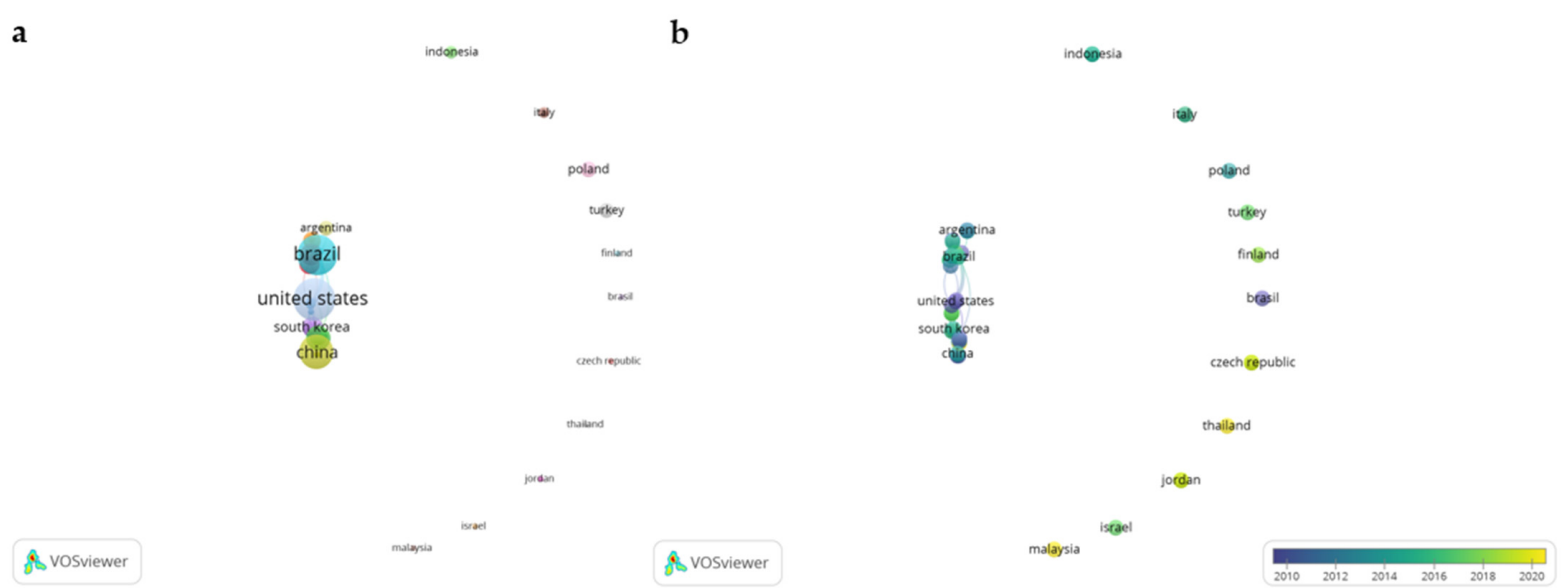
Network visualization map (**a**) and overlay visualization network (**b**) of countries with at least 1 paper on the field.

Regarding inter-country co-authorship, Figure 3a shows a network visualization map; it is similar to the overlay visualization map, but in this case, colored circles distinguish between groups according to the relationship between countries [31]. The group made up of the United States, Brazil, Argentina, South Korea, Argentina and China stands out. There is a strong link between the United States and Brazil, with a total link strength of 10 out of 19 for Brazil and 24 out of 24 for the United States. The other values of total link strength are less than or equal to three. Interestingly, there are 11 countries without international collaboration, although Italy (three), Indonesia (four), Turkey (five) and Poland (six) have published more than one paper. This information is of interest to build collaborations or explore new places to conduct research on the topic [51].

Figure 3b shows an overlay visualization network to define the participation of each country over time; the size of each circle was standardized for better visualization. The group of highlighted countries mentioned in the previous paragraph have contributed on average from 2008 (United States) to 2015 (Brazil). Countries with less participation and collaboration are relatively active in the field, having published papers in 2021 (Malaysia, Thailand); the change in the science is reaffirmed. It appears that in most (mainly high-income) countries where research began, it was determined that few causal mutations exist for tenderness traits in cattle. The techniques are being replicated in other countries with certain modifications (other CAST and CAPN isoforms, other genes, other cattle breeds, etc.). Genomic selection approaches were also adopted to analyze large numbers of SNPs distributed throughout the animal genome to estimate breeding values without requiring precise knowledge of gene location, [44].

#### 3.2.3. Most Prolific Institutions

USDA Agricultural Research Service is the institution with the most papers (21) and ranks second according to the TC/TP ratio (72.571). The University of Florida ranks ninth in Table 3 but has the highest TC/TP ratio (94.000). The number of papers/(TC/TP) ratio of the other countries is as follows: Universidade Estadual Paulista Júlio de Mesquita Filho 2:9, Empresa Brasileira de Pesquisa Agropecuária-Embrapa 3:8, Universidade de São Paulo 4:5, University of New England 5:6, Rural Development Administration 6:10, L’Institut national de la recherche agronomique 7:3, Universidade Federal de São Carlos 8:7 and CSIRO Livestock Industries 10:4. Institutions from Brazil (top 2, 37 papers) make up the majority of the top 10. All the institutions belong to the main countries shown in Table 2; therefore, the results are consistent.

#### 3.2.4. Most Prolific Journals

Table 4 lists the 10 journals with the most papers in the field of study. The *h* index proposed by Hirsch [87] is defined as an article whose order number (from highest to lowest according to the number of citations) is correlated to a lower or equal number of citations. The quartile number (Q) in which the journal is positioned in a specific field and the SCImago Journal Rank (SJR) index, which defines the quality and/or prestige of the journal according to the number of citations of the papers, were also determined [88]. Finally, the Journal Impact Factor (JIF) was determined; it measures the frequency with which the average number of articles in each journal has been cited during the previous two years [89].

The *Journal of Animal Science* was the journal with the most papers (28) and also with the highest TC/TP ratio (64.464). The number of papers/(TC/TP) ratio of the other journals was as follows: *Meat Science* 2:4, *Genetics and Molecular Research* 3:8, *Animal Genetics,* 4:3; *Animals,* 5:9; *Molecular Biology Reports,* 6:6; *Livestock Science,* 7:7; *Plos One,* 8:5; *Italian Journal of Animal Science,* 9:10 and *BMC Genetics* 10:2. *Genetics and Molecular Research, Animals, Plos One, Italian Journal of Animal Science* and *BMC Genetics* are exclusively open-access journals. Most of the journals are from the Netherlands and United Kingdom, and published by Elsevier. In addition, a large proportion of the journals, such as *Animals, Asian-Australasian Journal of Animal Science, Plos One, Animal,* and *BMC Genomics*, belong to Q1, but there are also many in Q2 (*Molecular Biology Reports, Animal Production Science, Archives Animal Breeding)*, Q3 (*African Journal of Biotechnology, BMC Genetics, Animal Biotechnology*) and Q4 (*Meta Gene, Journal of Central European Agriculture, Russian Journal of Genetics*).

The *Italian Journal of Animal Science* (42) and *Plos One* (367) had the lowest and highest H-index, respectively. *Genetics and Molecular Research* (0.583) and *Meat Science* (7.077) had the lowest and highest JIF, respectively. *Genetics and Molecular Research* (0.24) and *Meat Science* (1.3) had the lowest and highest SJR index, respectively. Wallin [90] suggests caution when evaluating journal quality with quantitative data. For example, the JIF favors journals whose papers have been widely cited in the last two years but then may not be cited, and a paper that has received few citations in the last two years may maintain that rate for several years. Perhaps for this reason, *Plos One* does not consider JIF as a metric. To reach a consensus, according to the study by Roldan-Valadez et al. [88], different metrics should be used in full to assess the influence and development of journals from different perspectives.

**Figure 4:**
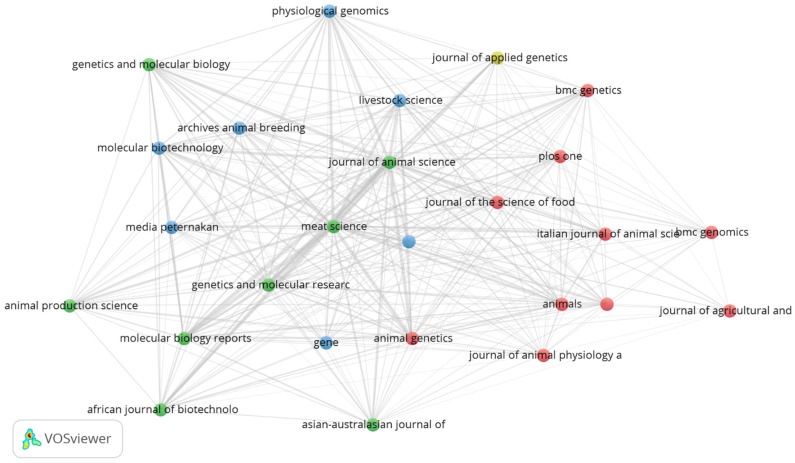
Bibliographic Coupling of journals with at least two papers.

Bibliographic coupling is a measure that indicates the relationship between two papers if they cite one or more papers in common [30]. As shown in Figure 4, most of the journals related to the topic have bibliographic coupling with varying intensity. The strength or intensity of bibliographic coupling is directly proportional to the similarity between the articles [19]. *Meat Science* and *Journal of Animal Science* have the highest total link strength (1654 and 1627, respectively), both linked (coupled) to 25 journals. *Genetic and Molecular Research* (24 links), *Molecular Biology Reports* (25 links) and *Animal Genetics* (25 links) have lower values of total link strength: 593, 585 and 414, respectively. The top 10 is completed by *Livestock Science* (25 links), *Physiological Genomics* (24 links), *Animals* (25 links), *Molecular Biotechnology* and *Genetics and Molecular Biology* (23 links) with total link strength of 414, 347, 334, 310 and 276, respectively.

### 3.3. Main Articles

The most influential papers according to the number of citations are shown in Table 5. The more citations a paper has, the more important it is considered to be for the evolution of a focal area [41]. The number of citations also depends on how many years ago a paper was published; therefore, this factor was also considered. The citations/citations per year ratio is as follows: Casas et al. [91] 1:5, Bernard et al. [61] 2:1, Casas et al. [64] 3:2, Page et al. [56] 4:6, White et al. [46] 5:7, Van Eenennaam et al. [92] 6:4, Page et al. [57] 7:9, Casas et al. [93] 8:10, Tizioto et al. [77] 9:3 and Allais et al. [67] 10:8. From the list of authors with the most contributions in the field (Table 1), Smith, T. P. L. (top 1) participated in the 3rd-7th most cited articles; Casas, E. (top 2) participated in the 1st, 3rd-5th and 7th-8th most cited articles; Keele, J. W. (top 4) participated in the 1st, 4th-5th and 7th most cited articles; Koohmaraie, M. (top 5) participated in the 1st, 3rd-5th and 7th most cited articles; Shackelford, S. D. (top 6) participated in the 1st, 3rd, 5th and 7th most cited articles; Coutinho, L. L (top 8) participated in the 9th most cited article; Wheeler, T. L. (top 9) participated in the 3rd-5th and 7th most cited articles and Bennett, G. L. (top 10) participated in the 7th-8th most cited articles.

A total of 70% of papers were published in the *Journal of Animal Science* (Top 1 in the ranking of journals in Table 4); 60% of papers [46,56,57,64,67,93] focused on the evaluation of SNPs in CAPN1 and CAST genes (in some cases) and their influence on beef tenderness. In addition, only one paper (rank 9) focused on genomic selection [77], the others used MAS. In general, a positive association of CAPN1 and a negative association of CAST on tenderness was found. This section of the paper confirms that studies on the relationship between meat tenderness with the calpain–calpastatin system and SNPs are predominant. For a better approach, Table 6 shows the most frequent keywords in the 175 papers retrieved from Scopus. If we focus on specific terms, the trend is to study the influence of calpastatin (46 occurrences) and calpain (41) with/without their SNPs (63) in cattle (37), specifically of the genus *Bos* (39). This is because proteolysis in muscle is the main factor contributing to tenderness [11]. In general, calpain and calpastatin participate in more than 40% of the variation in meat tenderness [61].

**Figure 5:**
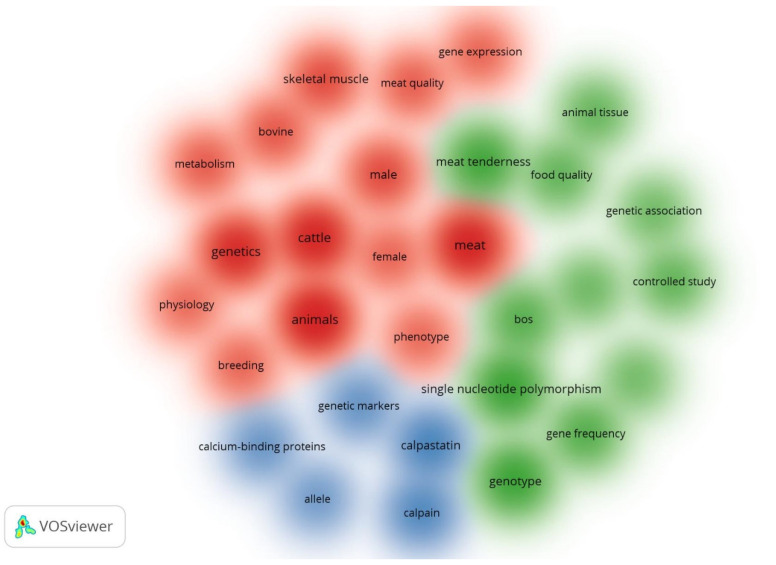
Cluster density visualization map of keywords with at least 25 occurrences.

To delimit the trend, Figure 5 shows the clusters of the most representative and most interconnected keywords. The cluster density visualization map is useful to have an overview on the classification of keywords according to whether they are related to each other; the color identifies the association between keywords [94]. Three groups of main keywords were obtained according to their correlation. The first group (blue) defines the role of calpain and calpastatin as genetic markers for the improvement of *Bos* cattle. It is recognized that postmortem meat tenderization is mainly due to endogenous enzymes that degrade proteins such as desmin, nebulin, troponin-T and titin [95]. The role of the calpain, cathepsin, caspases and proteasome enzyme systems has been documented [3,4,96]. The calpain system contributes up to 90% of proteolysis [97].

The calpain system includes at least three calcium-dependent isoforms, μ-calpain (CAPN1), m-calpain (CAPN2) and calpain 3 (originally named p94, CAPN3), and an inhibitor, calpastatin (CAST) [95]. Calpain 1 is considered the main enzyme because of its role in proteolysis in the early postmortem days, whereas calpain 2 acts later because in early postmortem, Ca^2+^ concentrations are lower than its requirement [95]. Therefore, calpain 2 may be more relevant in the later stages of aging [14]. On the other hand, although calpain 3 was reported not to significantly influence postmortem proteolysis of meat [10], promising results were obtained in some studies [98,99,100].

In the second group (green), SNPs explain the variability of meat tenderness. SNPs are common single-base variants of DNA, generally biallelic and easily detectable [21]. Variation in the calpain–calpastatin system genes was reported to affect the tenderness of different breeds and crossbreeds of cattle [61]. Therefore, SNPs are useful genetic markers to detect animals with superior traits [101]. The findings shown in Table 7 demonstrate the usefulness of SNPs and the importance of discovering other SNPs that explain variations in meat tenderness. It should be considered that the influence of SNPs may vary according to cattle breed. In the study by White et al. [46], the CAPN1 4751 and CAPN1 316 genes showed a strong association with the tenderness of the cattle (*B*. *indicus*, *B*. *taurus* and crossbreeds) evaluated. However, the CAPN1 530 marker was not associated with the tenderness of all cattle breeds. The authors suggest preferring markers that show association with a wider variety of populations. This group also has association with genomic selection. Lopes et al. [80] genotyped Nellore cattle with panels of 77K and 777K SNPs and successfully estimated breeding value for beef tenderness.

The third group (red) is confusing and not very specific; it can focus on the influence of gene expression on the meat (muscle) quality of cattle (bovines, specifically). The emphasis is again on beef. A total of 996.17 million head of cattle were reported worldwide [114]. Beef consumption was 6.377 kg per capita, higher than sheep meat consumption (1.766 kg per capita), but lower than pork and poultry meat consumption (10.817 and 14.858 kg per capita, respectively) [115]. By 2027, beef consumption is estimated to increase by 21% and 8% in developing and developed countries, respectively [116].

## 4. Conclusions

The study revealed an interest in the genes involved in meat tenderness. Over time, there were groups that conducted strong research in this field; they are currently inactive, but new research groups are being established. This is due to the evolution of science and new or updated methodologies for the selection and genetic improvement of livestock. According to the number of papers and/or citations, the United States and Brazil and their institutions lead the field individually and in interrelated studies. New research groups are replicating conventional studies in other countries, and another small group of papers deals with the use of new methodologies. However, it is of concern that there is little collaboration among the other countries. The *Journal of Animal Science* is the predominant journal, and together with the others, it has a high impact according to the different metrics evaluated, which guarantees the quality of the papers. The most influential articles, in addition to the trend and evolution of the main keywords, suggest that research is focused on CAPN and CAST genes and also SNPs due to their influence on beef tenderness. Likewise, genotyping (genomic selection) studies of a large number of SNPs for a better and faster explanation of the genetic variation of meat tenderness are on the rise.

## Figures and Tables

**Table 1:** Authors with more participation in research on the role of genetics in meat tenderness.

Ranking	Author	Publication Date Range	TP ^1^	Contribution (%) ^2^	Citations			
					TC ^3^	LL ^4^	UL ^5^	TC/TP
1	Smith, T. P. L.	1999–2019	13	7.429	944	11	183	72.615
2	Casas, E.	2000–2019	12	6.857	1197	13	202	99.750
3	de Oliveira, H. N.	2008–2019	12	6.857	252	1	57	21.000
4	Koohmaraie, M.	1995–2009	11	6.286	1088	14	202	98.909
5	Keele, J. W.	1999–2005	11	6.286	933	13	202	84.818
6	Shackelford, S. D.	1999–2019	10	5.714	890	13	202	89.000
7	Chardulo, L. A. L.	2008–2021	9	5.143	196	3	57	21.778
8	Wheeler, T. L.	2001–2019	8	4.571	748	13	183	93.500
9	Coutinho, L. L.	2013–2021	8	4.571	228	0	90	28.500
10	Bennett, G. L.	2001–2019	7	4.000	340	13	121	48.571

^1^ TP: total papers; ^2^ Contribution: TP/175 (papers retrieved) * 100; ^3^ TC: total citations; ^4^ LL: lower limit; ^5^ UL: upper limit.

**Table 2:** Countries with more participation in research on the role of causal genes in meat tenderness.

Ranking	Author	Publication Date Range	TP ^1^	Contribution (%) ^2^	Citations			
					TC ^3^	LL ^4^	UL ^5^	TC/TP
1	United States	1993–2021	41	23.429	1845	0	202	45.000
2	Brazil	2002–2021	37	21.143	688	0	90	18.595
3	China	2008–2021	27	15.429	204	0	22	7.556
4	Australia	2006–2020	14	8.000	426	10	64	30.429
5	Spain	2007–2020	11	6.286	136	0	42	12.364
6	France	2004–2020	10	5.714	448	9	187	44.800
7	South Korea	2008–2021	10	5.714	131	4	39	13.100
8	New Zealand	2000–2019	8	4.571	372	3	180	46.500
9	Canada	2010–2020	7	4.000	103	6	36	14.714
10	Poland	2004–2021	6	3.429	96	1	26	16.000

^1^ TP: total papers; ^2^ Contribution: TP/175 (papers retrieved) * 100; ^3^ TC: total citations; ^4^ LL: lower limit; ^5^ UL: upper limit.

**Table 3:** Institutions with more participation in research on the role of causal genes in meat tenderness.

Ranking	Author	Publication Date Range	TP ^1^	Contribution (%) ^2^	Citations			
					TC ^3^	LL ^4^	UL ^5^	TC/TP
1	USDA Agricultural Research Service	1992–2019	21	12.000	1524	11	202	72.571
2	Universidade Estadual Paulista Júlio de Mesquita Filho	2002–2021	17	9.714	321	1	57	18.882
3	Empresa Brasileira de Pesquisa Agropecuária-Embrapa	2012–2021	16	9.143	317	0	90	19.813
4	Universidade de São Paulo	2009–2021	13	7.429	343	0	90	26.385
5	University of New England	2007–2016	8	4.571	207	10	61	25.875
6	Rural Development Administration	2008–2019	8	4.571	110	4	39	13.750
7	L’Institut national de la recherche agronomique	2007–2020	6	3.429	317	9	187	52.833
8	Universidade Federal de São Carlos	2012–2021	6	3.429	144	0	90	24.000
9	University of Florida	2005–2018	5	2.857	470	9	183	94.000
10	CSIRO Livestock Industries	2006–2010	5	2.857	192	17	61	38.400

^1^ TP: total papers; ^2^ Contribution: TP/175 (papers retrieved) * 100; ^3^ TC: total citations; ^4^ LL: lower limit; ^5^ UL: upper limit.

**Table 4:** Journals with more research on the role of causal genes in meat tenderness.

Ranking	Journal	Publisher	Country	Q	Publication Date Range	TP ^1^	Contribution (%) ^2^	Citations				h Index ^6^	JIF ^7^ (2021)	SJR Index ^8^ (2021)
								TC ^3^	LL ^4^	UL ^5^	TC/TP			
1	*Journal of Animal Science*	Oxford University Press	United States	Q1	1995–2019	28	16.000	1805	9	202	64.464	164	3.338	0.85
2	*Meat Science*	Elsevier	Netherlands	Q1	2009–2019	16	9.143	322	4	65	20.125	175	7.077	1.3
3	*Genetics and Molecular Research*	Fundacao de Pesquisas Cientificas de Ribeirao Preto	Brazil	Q4	2011-2020	12	6.857	77	1	20	6.417	52	0.583	0.24
4	*Animal Genetics*	Wiley-Blackwell Publishing Ltd	United Kingdom	Q2	1999–2016	8	4.571	220	3	61	27.500	85	2.884	0.56
5	*Animals*	Multidisciplinary Digital Publishing Institute (MDPI)	Switzerland		2019–2021	6	3.429	21	1	6	3.500	43	3.231	0.61
6	*Molecular Biology Reports*	Springer	Netherlands	Q2	2011–2014	5	2.857	53	5	17	10.600	76	2.742	0.52
7	*Livestock Science*	Elsevier	Netherlands	Q1	2011–2019	5	2.857	45	1	19	9.000	116	1.929	0.52
8	*Plos One*	Public Library of Science	United States	Q1	2015–2016	4	2.286	62	6	37	15.500	367	-	0.85
9	*Italian Journal of Animal Science*	Taylor and Francis	United Kingdom	Q2	2007–2020	4	2.286	11	0	6	2.750	42	2.552	0.55
10	*BMC Genetics*	BioMed Central	United Kingdom	Q3	2008–2019	3	1.714	117	24	55	39.000	80	2.759	0.59

^1^ TP: total papers; ^2^ Contribution: TP/175 (papers retrieved) * 100; ^3^ TC: total citations; ^4^ LL: lower limit; ^5^ UL: upper limit; ^6^ h index: Hirsch index; ^7^ JIF: Journal Impact Factor; ^8^ SJR: SCImago Journal Rank.

**Table 5:** Most cited articles on the field of study.

Ranking	Reference	Year of Publication	Number of Authors	Title	Journal	Citations	Citations Per Year
1	Casas et al. [91]	2000	6	Quantitative trait loci affecting growth and carcass composition of cattle segregating alternate forms of myostatin	*Journal of Animal Science*	202	9.182
2	Bernard et al. [61]	2007	6	New Indicators of Beef Sensory Quality Revealed by Expression of Specific Genes	*Journal of Agricultural and Food Chemistry*	187	12.467
3	Casas et al. [64]	2006	9	Effects of calpastatin and μ-calpain markers in beef cattle on tenderness traits	*Journal of Animal Science*	183	11.438
4	Page et al. [56]	2002	11	Evaluation of single-nucleotide polymorphisms in CAPN1 for association with meat tenderness in cattle	*Journal of Animal Science*	180	9.000
5	White et al. [46]	2005	10	A new single nucleotide polymorphism in CAPN1 extends the current tenderness marker test to include cattle of *Bos indicus*, *Bos taurus*, and crossbred descent	*Journal of Animal Science*	150	8.824
6	Van Eenennaam et al. [92]	2007	8	Validation of commercial DNA tests for quantitative beef quality traits	*Journal of Animal Science*	149	9.933
7	Page et al. [57]	2004	12	Association of markers in the bovine CAPN1 gene with meat tenderness in large crossbred populations that sample influential industry sires	*Journal of Animal Science*	121	6.722
8	Casas et al. [93]	2005	10	Assessment of single nucleotide polymorphisms in genes residing on chromosomes 14 and 29 for association with carcass composition traits in *Bos indicus* cattle	*Journal of Animal Science*	114	6.706
9	Tizioto et al. [77]	2013	21	Genome scan for meat quality traits in Nelore beef cattle	*Physiological Genomics*	90	10.000
10	Allais et al. [67]	2011	11	Effects of polymorphisms in the calpastatin and µ-calpain genes on meat tenderness in 3 French beef breeds	*Journal of Animal Science*	75	6.818

**Table 6:** Keywords with the highest occurrence in the papers.

Ranking	Keyword ^1^	Occurrence	Ranking	Keyword	Occurrence
1	article	100	11	calpastatin	46
2	animals	97	12	skeletal muscle	45
3	meat	88	13	gene frequency	41
4	cattle	85	14	calpain	41
5	genetics	77	15	bos	39
6	single nucleotide polymorphism	63	16	bovine	37
7	genotype	62	17	metabolism	35
8	nonhuman	62	18	meat quality	34
9	meat tenderness	55	19	controlled study	33
10	male	46	20	food quality	33

^1^ Related keywords such as animal (84 occurrences) and animalia with (37) were omitted.

**Table 7:** Studies on the role of CAPN and CAST genes and their SNPs on meat tenderness (measured by shear force tests) in cattle.

Reference	Population	Muscle ^1^	Genes ^2^	
			CAPN	CAST
[46]	Brahman, *B*. *taurus*, and germplasm from *B*. *indicus* and *B*. *taurus*	*Longissimus*	CAPN1 316^+^, CAPN1 4753^+^ and CAPN1 530^+^	
[64]	*B*. *indicus* and *B*. *taurus*	N.S.	CAPN1^+^	CAST^+^
[56]	Piedmontese × Angus and Jersey × Limousin	*Longissimus thoracis*	38 SNPs^+^	
[102]	Jersey-Limousin crosses, Angus and Hereford-cross	*Longissimus dorsi*	CAPN1: c.947C > G^+^	CAST: c.2959A > G^+^
[60]	Santa Gertrudis, Brahman and Belmont Red	*Longissimus lumborum*	CAPN3:c.2443-103G > C^+^, CAPN3:c.53T>G^+^ and CAPN3:c.1538+225G > T^+^	CAST:c.2832A > G^+^
[103]	Brahman	*Longissimus dorsi*	CAPN316^+^ and CAPN4751^+^	CAST^+^
[104]	Nellore	*Longissimus dorsi*	CAPN1 316^+^, CAPN1 4751^+^, CAPN1 530^+^ and CAPN1 4753^+^	UOGCAST^+^ and WSUCAST^+^
[105]	Nellore	*Longissimus dorsi*	CAPN1 4751^−^	
[106]	Charolais, Limousin and Retinta	*Longissimus dorsi*	CAPN1^+^	CAST^+^
[70]	Nellore	*Longissimus dorsi*	CAPN1^−^ and CAPN2^-^	CAST^−^
[107]	Parda de Montaña and Pirenaica	*Longissimus thoracis*	CAPN1 316^−^, CAPN1 530^−^ and CAPN1 4751^−^	CAST1^+^, CAST2^+^, CAST3^−^, CAST4^+^ and CAST5^−^
[108]	Hanwoo	*Longissimus lumborum*	CAPN1:c.1589G > A^+^, CAPN1:c.658C > T^+^, CAPN1:c.948G > C^+^ and CAPN1:c.580A, > G^+^	CAST:c.182A > G^+^, CAST:c.1985G > C^+^ and CAST:c.1526T > C^+^
[109]	*B*. *taurus*, *B*. *indicus* and crosses	*Longissimus dorsi*	CAPN1 316^+^ and CAPN1 4751^+^	CAST-T1^−^
[110]	Nellore	*Longissimus dorsi*	CAPN1^−^ and CAPN2^−^	CAST^−^
[72]	Nellore	*Longissimus thoracis*	CAPN1^−^ and CAPN2^−^	CAST1^−^ and CAST2^+^
[111]	Turkish grey	*Longissimus dorsi*	CAPN1 316^+^ and CAPN1 4751^+^	UOGCAST^+^
[112]	Nelore	*Longissimus thoracis*	CAPN1 4751^+^	UOGCAST^+^
[113]	Angus, Charolais, Brahman and Nguni	*Longissimus thoracis et lumborum*	CAPN1 184^+^, CAPN1 187^+^, CAPN1 4751^+^ and CAPN2 780^+^	CAST 736^+^ and CAST 763^+^

^1^ N.S.: not specified; ^2^ without association with meat tenderness (−) and with association with meat tenderness (+).

## Data Availability

Not applicable.
